# Supplementary material for: Complexome profiling of the Chlamydomonas psb28 mutant reveals TEF5 as an early PSII assembly factor
Source: Plant Cell. 2025 Mar 17;37(6):koaf055. doi: 10.1093/plcell/koaf055 (PMC12164590; doi:10.1093/plcell/koaf055)
Supplement: koaf055_Supplementary_Data [file koaf055_supplementary_data.zip › psb28_interactive.zip!/plots/intensity/qualityPlots/peptides/_Cre14.g612800.t1.2.html]

### Cre14.g612800.t1.2

Trivial name:   
SeqCoverage [median]: 45.00  
Mass: 16.36 kDa  
  
 

| Sequence | MissedCleavage | Mass [Da] | wtIntensity | muIntensity | Score |
| --- | --- | --- | --- | --- | --- |
| ASFGVYQAPPPVGPVESVQNGVQDLLER | 0 | 2952.50 | 1923.98 | 0.00 | 82.65 |
| DGPLAVEEVAVDEFNAPTKR | 1 | 2156.07 | 25059.61 | 52999.84 | 145.05 |
| TLQELGFTEQAADILAFSK | 0 | 2081.07 | 10716.65 | 0.00 | 123.43 |
|  |  |  | 37700.2 | 52999.8 |  |
